# Association Between Overweight/Obesity Metabolic Phenotypes Defined by Two Criteria of Metabolic Abnormality and Cardiovascular Diseases: A Cross‐Sectional Analysis in a Chinese Population

**DOI:** 10.1002/clc.70020

**Published:** 2024-10-14

**Authors:** Yue Qiu, Shujin Fan, Jing Liu, Xiaodan He, Tianxin Zhu, Li Yan, Meng Ren

**Affiliations:** ^1^ Department of Endocrinology, Sun Yat‐sen Memorial Hospital Sun Yat‐sen University Guangzhou China; ^2^ Guangdong Clinical Research Center for Metabolic Diseases Guangzhou Key Laboratory for Metabolic Diseases Guangzhou China

**Keywords:** cardiovascular disease, metabolic, obesity, phenotype

## Abstract

**Objectives:**

Obesity/overweight and metabolic anomalies are known to be associated with elevated cardiovascular disease (CVD) risk. However, there is a paucity of research exploring the association between different body weights, varying metabolic statuses, and the occurrence of CVD in the Chinese population. Thus, we performed this study to explore the relation between different metabolic overweight/obesity phenotypes and the prevalence of CVD.

**Methods:**

We analyzed data from 9075 participants in the Risk Evaluation of cAncers in Chinese diabeTic Individuals: A lONgitudinal (REACTION) study. Participants were classified into four metabolic phenotypes based on their metabolic status and obesity/overweight status. Regression analysis was used to evaluate the relationship between CVD and different groups. Additionally, we conducted a subgroup analysis to further explore the relationship between CVD and different metabolic abnormalities.

**Results:**

Compared to metabolically healthy non‐overweight/obesity (MHNO) individuals, both overweight/obesity and metabolic anomalies were positively associated with CVD prevalence. Among other metabolically unhealthy and overweight/obesity phenotypes, metabolically healthy overweight/obesity (MHO) generally exhibited a comparatively lower association with CVD. In the elderly, high waist circumference was significantly associated with CVD, rather than body weight. Further analysis revealed that hypertension had the strongest association with CVD.

**Conclusion:**

Elderly individuals should place more emphasis on managing their waist circumference rather than only on BMI. CVD prevention should focus on both body weight management and treatment of metabolic diseases, with particular emphasis on antihypertensive therapy.

## Summary

- Our study used two types of criteria to define metabolic unhealthiness and explored the association between different metabolic overweight/obesity phenotypes and CVD.
- In the elderly, high waist circumference, rather than on high BMI, was associated with CVD.
- Hypertension had the strongest association with CVD compared to other metabolic anomalies.

## Graphical abstract

Our study explored the association between different metabolically overweight/obesity phenotypes and CVD in the Chinese population. The results demonstrated that elderly individuals should place more emphasis on managing their waist circumference rather than only on BMI. CVD prevention should focus on both body weight management and treatment of metabolic disease, with particular emphasis on antihypertensive therapy.

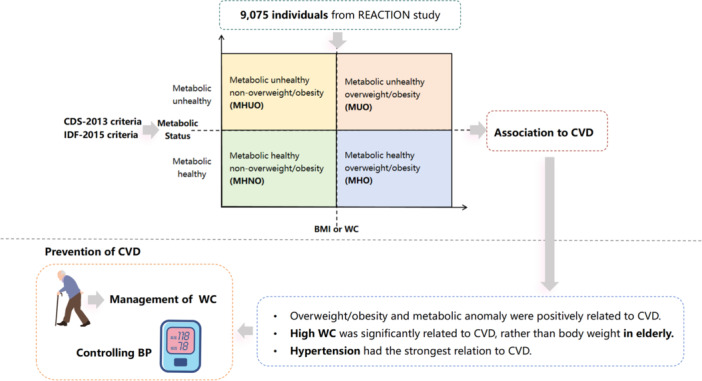

**Abbreviations**
ASCVD: atherosclerotic cardiovascular disease
BMI: body mass index
CDS‐2013: China Diabetes Society 2013 guideline
CHOL: total cholesterol
CVD: cardiovascular disease
DBP: diastolic blood pressure
FBG: fasting blood glucose
GBD: Global Burden of Disease study
HDL‐C: high‐density lipoprotein cholesterol
HOMA‐IR: the homeostasis model assessment of insulin resistance
IDF‐2005: International Diabetes Federation 2005 guideline
LDL‐C: low‐density lipoprotein cholesterol
MHNO: metabolically healthy non‐overweight/obesity
MHO: metabolically healthy overweight/obesity
MUNO: metabolically unhealthy non‐overweight/obesity
MUO: metabolically unhealthy overweight/obesity
NCEP: US National Cholesterol Education Program
OGTT‐2 h: oral glucose tolerance test 2 h
REACTION: the Risk Evaluation of cAncers in Chinese diabeTic Individuals: A lONgitudinal study
SBP: systolic blood pressure
TG: triglycerides
WC: waist circumference
WHO: World Health Organization

## 1 Introduction

Cardiovascular disease (CVD) is the leading cause of mortality worldwide. According to 2019 Global Burden of Disease (GBD) study, CVD was responsible for 18.6 million deaths, accounting for over 40% of non‐communicable diseases. This imposes a substantial financial burden on healthcare systems and society [1, 2, 3]. Among various countries, China had the highest disease burden, with CVD contributing to 40% mortality in the Chinese population [4]. Thus, it is of great significance to identify the risk factors for CVD so that early intervention and prevention strategies can be implemented for populations at risk.

Overweight and obesity, recognized as serious health issues, affect more than 10% of adults worldwide and are widely acknowledged to be associated with CVD [5]. Although overweight and obesity always coexist with dyslipidemia, insulin resistance, and hypertension [6], not all obesity phenotypes present equally, leading to different metabolic obesity phenotypes. In addition to metabolically unhealthy obesity (MUO) individuals, there is a substantial number of obese individuals classified as metabolically healthy obesity (MHO), who do not exhibit metabolic anomalies [7, 8]. Several studies have reported that different metabolic obesity phenotypes are associated with varying risks and mortality rates for CVD [9, 10]. It appears that utilizing different metabolic profiles to stratify obesity can provide more accurate predictions to the risk of diseases and highlight specific phenotypes, thereby offering valuable insights for clinical intervention and prevention strategies.

Due to the use of various criteria for metabolic abnormalities across different studies, as well as differences in age, sex, and ethnicity, the reported prevalence of MHO has shown considerable variability [11, 12]. Additionally, current methods for assessing overweight and obesity include different indicators such as body mass index (BMI) and waist circumference (WC). However, the thresholds for normal weight and WC vary by regions and populations [13, 14], resulting in increased heterogeneity when categorizing distinct metabolic obesity phenotypes.

There are few studies investigating the relationship between overweight/obesity, stratified by metabolic status, and the incidence of CVD in the Chinese population. Thus, we analyzed data from Risk Evaluation of cAncers in Chinese diabeTic Individuals: A lONgitudinal (REACTION) study, which utilized different definitions of metabolic abnormalities and overweight/obesity, to explore the relationship between different metabolic overweight/obesity phenotypes and CVD.

## 2 Method

### 2.1 Study Populations

**Figure 1:**
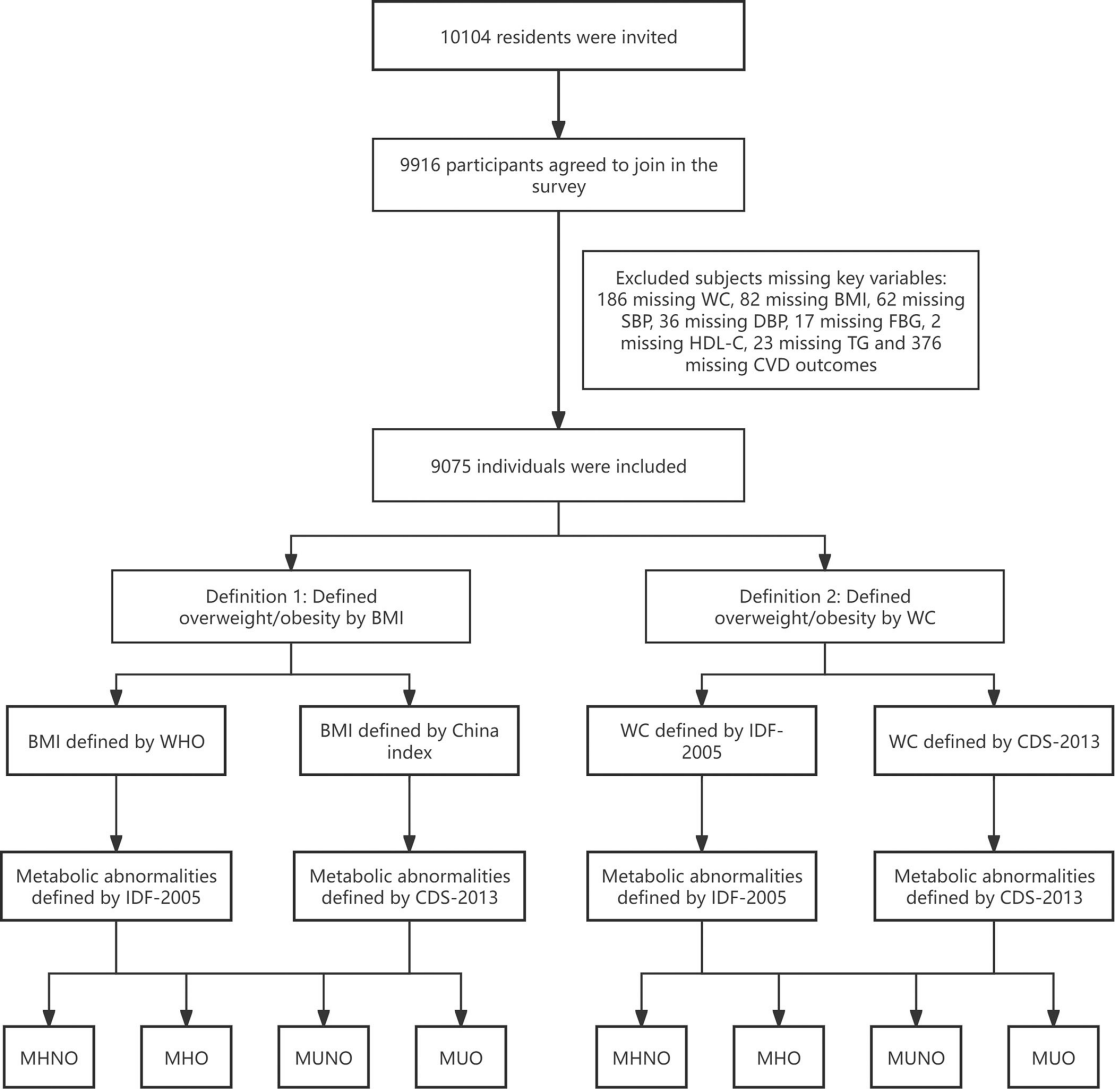
Flowchart for selection of study participants. BMI, body mass index; CVD, cardiovascular disease; DBP, diastolic blood pressure; FBG, fasting blood glucose; HDL‐C, high‐density lipoprotein cholesterol; MHNO, metabolically healthy non‐overweight/obesity; MHO, metabolic healthy overweight/obesity; MUNO, metabolically unhealthy non‐overweight/obesity; MUO, metabolic unhealthy overweight/obesity; SBP, systolic blood pressure; TG, triglycerides; WC, waist circumference.

The study data were extracted from the REACTION study, which is a multicenter, prospective observational study that aims to evaluate chronic diseases in the Chinese population [15, 16]. The study population consisted of residents from communities in Guangzhou, China, from June to November 2011. During the recruitment phase, a total of 10 104 residents aged 40 years or older were invited to participate through examination notices or home visits. A total of 9916 subjects signed the consent form and agreed to participate in the survey. Subsequently, subjects with missing key variables were excluded, including 186 subjects missing WC, 82 subjects missing BMI, 62 missing systolic blood pressure (SBP), 36 missing diastolic blood pressure (DBP), 17 missing fasting blood glucose (FBG), 2 missing high‐density lipoprotein cholesterol (HDL‐C), 23 missing triglycerides (TGs), and 376 missing the history of CVD. Ultimately, a total of 9075 eligible individuals were included in the data analyses. This study was approved by the Ethics Committee of Sun Yat‐sen Memorial Hospital, Sun Yat‐sen University. The ethics approval number is Sun Yat‐sen Memorial Hospital of Zhongshan University [2019] Ethical Approval Research No. 38. A detailed inclusion and exclusion process is shown in Figure 1.

### 2.2 Measurements

Anthropometric assessments were conducted by well‐trained staff using standardized procedures. A comprehensive questionnaire was administered, collecting information on age, gender, past medical history, present illness, history of drug supplementation, current drinking habits, and current smoking habits. Measurements of body height and weight were taken with subjects wearing light clothing and without shoes. BMI was calculated as weight/height squared (kg/m^2^). Following a 5‐min rest period, SBP and DBP were measured three times by using an automated electronic device (OMRON Model HEM‐752 FUZZY; Omron Company). The average of the three measurements of blood pressure were used for analysis.

During laboratory tests, whole blood samples were collected after an overnight fast of at least 10 h. TG, total cholesterol (CHOL), low–density lipoprotein cholesterol (LDL‐C), HDL‐C, FBG, oral glucose tolerance test 2 h (OGTT‐2 h), and fasting insulin were measured using an autoanalyzer (Beckman CX‐7 Biochemical Autoanalyzer, Brea, CA, USA). Hemoglobin A1c (HbA1c) was measured by high‐performance liquid chromatography (Bio‐Rad, Hercules, CA, USA). Fasting insulin was measured using an autoanalyzer (ARCHITECT i2000SR System, Abbott Laboratories). The homeostasis model assessment of insulin resistance (HOMA‐IR) was calculated as fasting insulin (μIU/mL) × fasting blood glucose (mmol/L)/22.5 [17].

### 2.3 Definition of Metabolic Disorders, Obesity, and CVD

The metabolic disorders were defined using the criteria of the International Diabetes Federation in 2005 (IDF‐2005) and the China Diabetes Society 2013 guideline (CDS‐2013) separately [18, 19, 20]. According to IDF‐2005, metabolic disorders were defined as follows: (1) Hypertension: SBP ≥ 130 mmHg and/or DBP ≥ 85 mmHg or treatment of previously diagnosed hypertension. (2) Hyperglycemia: FBG ≥ 5.6 mmol/L and/or previously diagnosed Type 2 diabetes. (3) Dyslipidemia: TG ≥ 1.7 mmol/L and/or HDL‐C < 1.03 mmol/L for men, < 1.29 mmol/L for women, or treatment for lipid abnormality. According to CDS‐2013, metabolic disorders were defined as follows: (1) Hypertension: SBP ≥ 130 mmHg and/or DBP ≥ 85 mmHg or treatment of previously diagnosed hypertension. (2) Hyperglycemia: FBG ≥ 5.6 mmol/L and/or OGTT‐2 h ≥ 7.8 mmol/L or previously diagnosed Type 2 diabetes. (3) Dyslipidemia: TG ≥ 1.7 mmol/L and/or HDL‐C < 1.03 mmol/L or treatment for lipid abnormality. Detailed definitions are shown in Supporting Information S1: Table S1.

Overweight/obesity was defined using two criteria as follows: Definition 1, based on BMI, and Definition 2, based on WC. Since WC reflects visceral adiposity, which is strongly associated with metabolic complications, it was also used [21]. When using Definition 1, in the IDF‐2005 criteria, obesity/overweight was defined as BMI ≥ 25 kg/m^2^, according to World Health Organization (WHO); in the CDS‐2013 criteria, obesity/overweight was defined as BMI ≥ 24 kg/m^2^, according to Working Group on Obesity in China [13]. When using Definition 2, in the IDF‐2005 criteria, overweight/obesity was defined as WC ≥ 90 cm for men and ≥ 80 cm for women, whereas in the CDS‐2013 criteria, overweight/obesity was defined as WC ≥ 90 cm for men and ≥ 85 cm for women, according to the definition of their specific definition of abdominal obesity. The history of CVD was self‐reported and obtained from the standard questionnaire, which was reviewed and confirmed by researchers. The CVD events were defined as history of coronary heart disease, myocardial infarction, stroke, transient ischemic attack, or intervention for peripheral artery disease [22, 23].

### 2.4 Definitions of Different Metabolic Overweight/Obesity Phenotypes

Participants were divided into four groups according to their metabolic disorders and overweight/obesity status [24] as follows: (1) Metabolically healthy non‐overweight/obesity (MHNO): Normal weight with ≤ 1 type of metabolic disorders. (2) Metabolically healthy overweight/obesity (MHO): Overweight/obesity with ≤ 1 type metabolic disorders. (3) Metabolically unhealthy non‐overweight/obesity (MUNO): Normal weight with ≥ 2 types of metabolic disorders. (4) Metabolic unhealthy overweight/obesity (MUO): Overweight/obesity with ≥ 2 types of metabolic disorders.

### 2.5 Further Subgroups of Metabolic Disorders

To further investigate the correlation between different combinations of metabolic abnormalities and the onset of CVD across various weight statuses, we categorized the metabolic abnormalities in the following ways: (1) Metabolically healthy: Without any metabolic disorders. (2) Single hypertension: Presence of hypertension only. (3) Single hyperglycemia: Presence of blood glucose disorders only. (4) Single dyslipidemia: Presence of lipid disorders only. (5) Hypertension accompanied by hyperglycemia: Presence of both hypertension and blood glucose disorders simultaneously. (6) Hypertension accompanied by dyslipidemia: Presence of both hypertension and lipid disorders simultaneously. (7) Hyperglycemia accompanied by dyslipidemia: Presence of both blood glucose disorders and lipid disorders simultaneously. (8) Hypertension accompanied by hyperglycemia and dyslipidemia: Presence of hypertension, blood glucose disorders, and lipid disorders simultaneously.

### 2.6 Statistical Analysis

The prevalence of CVD, demographics, and characteristics of subjects were compared across different metabolic overweight/obesity phenotypes groups. The continuous data were expressed by mean ± SD, whereas the categorical data were expressed as numbers (percentage). Differences among groups were tested using ANOVA for continuous data and the chi‐squared test for categorical data. Multivariate logistic regression was used to identify the correlation between CVD and different overweight/obesity phenotypes groups when using MHNO as the reference. Model 1 was non‐adjusted, whereas Model 2 was adjusted for age, gender, drinking, and smoking habits. Since age is one of the critical factors that affect CVD [25, 26], we stratified the population by age and used multivariate logistic regression to analyze the correlation between CVD and different overweight/obesity phenotypes in different age groups. Finally, we conducted regression analysis on subgroups of different metabolic disorders stratified by BMI or WC. A two‐sided *p* < 0.05 was considered significant. Data were analyzed using the statistical packages R (The R Foundation; http://www.r-project.org, Version 4.2.0) and EmpowerStats (https://www.empowerstats.net, X&Y Solutions Inc. Boston, MA, USA).

## 3 Result

### 3.1 Characteristics of Subjects Across Different Metabolic Overweight/Obesity Phenotypes

The general characteristics of subjects are shown in Table 1. The general characteristics were examined in 9075 subjects, with a mean age of 55.89 ± 8.03 years, of whom 71.79% were female. Among all subjects, 255 (2.81%) individuals had a history of CVD, while 23.18% had a drinking habit, and 12.30% were smokers. Subjects were categorized into four groups based on the IDF‐2005 or CDS‐2013 criteria as defined in Definition 1 (Supporting Infomation S1: Table S2). Comparisons revealed that individuals classified as metabolically unhealthy tended to be older, smokers, and exhibited elevated levels of SBP, DBP, FBG, TG, and OGTT‐2H, regardless of whether the IDF‐2005 or CDS‐2013 criteria were applied. Moreover, according to Definition 1, individuals categorized as overweight or obese tended to have higher WC levels. In Definition 2, when using WC to define overweight/obesity, similar trends were observed in age and various biochemical measurements among individuals as in Definition 1 (Supporting Infomation S1: Table S3). Additionally, participants classified as overweight or obese based on WC also had exhibited higher BMI, surpassing normal BMI range.

**Table 1:** General characteristics of participants.

Characteristics	Total (*n* = 9075)
Age (years)	55.89 ± 8.03
Waist circumference (cm)	81.62 ± 9.47
Body mass index (kg/m^2^)	23.64 ± 3.39
Height (cm)	158.27 ± 7.64
Weight (kg)	59.29 ± 9.76
Systolic blood pressure (mmHg)	126.02 ± 16.53
Diastolic blood pressure (mmHg)	75.28 ± 9.87
Fasting blood glucose (mmol/L)	5.68 ± 1.34
OGTT‐2 h (mmol/L)	8.08 ± 3.09
Total cholesterol (mmol/L)	1.58 ± 1.20
HDL‐cholesterol (mmol/L)	1.32 ± 0.36
LDL‐cholesterol (mmol/L)	3.14 ± 0.96
Total triglycerides (mmol/L)	5.19 ± 1.25
Hemoglobin A1c (%)	6.05 ± 0.9
Fasting insulin (μIU/mL)	8.26 ± 5.85
HOMA‐IR	2.15 ± 1.97
Cardiovascular diseases (%)	
No	8820 (97.19%)
Yes	255 (2.81%)
Gender (%)	
Female	6515 (71.79%)
Male	2560 (28.21%)
Drinking (%)	
No	6884 (76.82%)
Yes	2077 (23.18%)
Smoking (%)	
No	7856 (87.70%)
Yes	1102 (12.30%)

*Note:* All data are reported as mean ± SD and absolute frequencies were proper.

Abbreviations: HDL, high‐density lipoprotein; HOMA‐IR, homeostasis model assessment of insulin resistance; LDL, low‐density lipoprotein; OGTT‐2 h, oral glucose tolerance test 2 h.

### 3.2 The Association Between CVD and Overweight/Obesity Metabolic Phenotypes

Table 2 showed the relations between the incidence of CVD and different overweight/obesity phenotypes. Based on the IDF‐2005 criteria, without statistical adjustment, there was no significant association between MHO and onset of CVD, as evidenced in both Definition 1 (OR = 1.58, 95% CI 0.96–2.60, *p* = 0.07) and Definition 2 (OR = 1.49, 95% CI 0.99–2.23, *p* = 0.06). Both MUNO (OR = 1.66, 95% CI 1.17–2.35, *p* < 0.01) and MUO (OR = 2.44, 95% CI 1.73–3.43, *p* < 0.0001) showed a positive correlation with the onset of CVD in Definition 1, and a similar trend was observed in Definition 2. These trends remained consistent after adjustments were made. However, using the CDS‐2013 criteria, the analysis revealed a consistent positive correlation between both overweight/obesity and metabolic unhealthy phenotypes with CVD, across both Definition 1 and Definition 2. Specifically, under Definition 1, after adjustment, the association of MUNO with CVD (OR = 2.01, 95% CI 1.34–3.01, *p* < 0.01) was stronger compared to that of MHO (OR = 1.61, 95% CI 1.06–2.45, *p* < 0.05). Conversely, in Definition 2, after adjustment, the association of MUNO with CVD (OR = 1.59, 95% CI 1.13–2.25, *p* < 0.01) was weaker compared to that of MHO (OR = 1.75, 95% CI 1.15–2.68, *p* < 0.01).

**Table 2:** The association between CVD and different groups of metabolic phenotypes defined by the IDF criteria and CDS criteria.

	Model 1		Model 2	
	OR (95% CI)	*p* value	OR (95% CI)	*p* value
IDF‐2005 criteria				
Definition 1				
MHNO	1		1	
MHO	1.58 (0.96, 2.60)	0.07	1.66 (1.00, 2.74)	0.05
MUNO	1.66 (1.17, 2.35)	< 0.01	1.47 (1.03, 2.10)	< 0.05
MUO	2.44 (1.73, 3.43)	< 0.0001	2.22 (1.57, 3.15)	< 0.0001
Definition 2				
MHNO	1		1	
MHO	1.49 (0.99, 2.23)	0.06	1.49 (0.98, 2.26)	0.06
MUNO	1.85 (1.26, 2.73)	< 0.01	1.64 (1.10, 2.43)	< 0.05
MUO	2.23 (1.58, 3.15)	< 0.0001	1.99 (1.39, 2.86)	< 0.01
CDS‐2013 criteria				
Definition 1				
MHNO	1		1	
MHO	1.61 (1.06, 2.44)	< 0.05	1.61 (1.06, 2.45)	< 0.05
MUNO	2.03 (1.36, 3.04)	< 0.01	2.01 (1.34, 3.01)	< 0.01
MUO	3.36 (2.38, 4.75)	< 0.0001	3.26 (2.30, 4.62)	< 0.0001
Definition 2				
MHNO	1		1	
MHO	1.88 (1.24, 2.84)	< 0.01	1.75 (1.15, 2.68)	< 0.01
MUNO	2.30 (1.65, 3.20)	< 0.0001	1.59 (1.13, 2.25)	< 0.01
MUO	3.04 (2.17, 4.26)	< 0.0001	1.97 (1.38, 2.80)	< 0.01

*Note:* Model 1: Unadjusted. Model 2: Adjusted for age, gender, drinking, and smoking habits. Definition 1: Obesity defined by BMI. Definition 2: Obesity defined by WC instead of BMI. IDF criteria: Metabolic disorders defined by 2005 International Diabetes Federation criteria. CDS criteria: Metabolic disorders defined by 2013 Chinese Diabetes Society criteria.

Abbreviations: MHNO, metabolically healthy non‐overweight/obesity; MHO, metabolically healthy overweight/obesity; MUNO, metabolically unhealthy non‐overweight/obesity; MUO, metabolically unhealthy overweight/obesity; OR, odds ratio.

*p* value < 0.05 indicates significance.

### 3.3 Association between CVD and Overweight/Obesity Metabolic Phenotypes Across Different Age Groups

As shown in Table 3, individuals were categorized into two age subgroups: 40–60 years old and 60–80 years old. For the 40–60 years subgroup, analysis using the IDF‐2005 criteria revealed no significant correlation between CVD and MHO under Definition 1. Under Definition 2, no significant correlation were observed between CVD and both MHO and MUNO. Similar results were observed when applying the CDS‐2013 criteria. In the 60–80 years subgroup, under Definition 1, MHO was not significantly associated with CVD according to both the IDF‐2015 and CDS‐2013 criteria. Conversely, under Definition 2, and regardless of whether IDF‐2005 or CDS‐2013 criteria were applied, all phenotypes of overweight/obesity and metabolic abnormalities showed a significant positive correlation with CVD (all *p* < 0.05 or *p* < 0.01).

**Table 3:** The association between CVD and different groups of metabolic phenotypes defined by the IDF criteria and CDS criteria, stratified by age.

	Non‐adjusted		Adjusted			Non‐adjusted		Adjust	
	OR (95% CI)	*p* value	OR (95% CI)	*p* value		OR (95% CI)	*p* value	OR (95% CI)	*p* value
IDF‐2005 criteria					CDS‐2013 criteria				
Age (40, 60)					Age (40, 60)				
Definition 1					Definition 1				
MHNO	1		1		MHNO	1		1	
MHO	1.17 (0.56, 2.46)	0.68	1.31 (0.62, 2.78)	0.48	MHO	1.46 (0.82, 2.59)	0.20	1.44 (0.80, 2.58)	0.23
MUNO	1.72 (1.02, 2.92)	< 0.05	1.35 (0.78, 2.32)	0.28	MUNO	1.36 (0.71, 2.60)	0.35	1.38 (0.71, 2.66)	0.34
MUO	2.47 (1.51, 4.03)	< 0.01	2.38 (1.44, 3.92)	< 0.01	MUO	2.65 (1.61, 4.38)	< 0.01	2.69 (1.62, 4.48)	< 0.01
Definition 2					Definition 2				
MHNO	1		1		MHNO	1		1	
MHO	1.08 (0.60, 1.93)	0.80	1.13 (0.62, 2.04)	0.70	MHO	1.07 (0.56, 2.04)	0.84	1.12 (0.58, 2.14)	0.74
MUNO	1.55 (0.86, 2.80)	0.15	1.20 (0.65, 2.22)	0.56	MUNO	1.62 (1.00, 2.63)	0.05	1.66 (1.00, 2.72)	0.05
MUO	2.31 (1.42, 3.75)	< 0.01	2.10 (1.28, 3.46)	< 0.01	MUO	1.91 (1.14, 3.20)	< 0.05	1.94 (1.15, 3.27)	< 0.05
Age (60, 80)					Age (60, 80)				
Definition 1					Definition 1				
MHNO	1		1		MHNO	1		1	
MHO	2.04 (1.00, 4.15)	0.05	2.02 (0.98, 4.14)	0.06	MHO	1.80 (0.96, 3.37)	0.07	1.81 (0.96, 3.40)	0.07
MUNO	1.70 (1.05, 2.76)	< 0.05	1.50 (0.92, 2.46)	0.11	MUNO	1.52 (0.86, 2.67)	0.15	1.50 (0.85, 2.63)	0.16
MUO	2.52 (1.54, 4.13)	< 0.01	2.17 (1.31, 3.59)	< 0.01	MUO	2.66 (1.60, 4.41)	< 0.01	2.54 (1.52, 4.23)	< 0.01
Definition 2					Definition 2				
MHNO	1		1		MHNO	1		1	
MHO	2.22 (1.21, 4.06)	< 0.01	2.12 (1.14, 3.95)	< 0.05	MHO	2.80 (1.54, 5.10)	< 0.01	2.76 (1.51, 5.03)	< 0.01
MUNO	2.26 (1.30, 3.94)	< 0.01	2.03 (1.16, 3.56)	< 0.05	MUNO	1.93 (1.17, 3.20)	< 0.05	1.85 (1.11, 3.07)	< 0.05
MUO	2.43 (1.45, 4.08)	< 0.01	2.04 (1.18, 3.51)	< 0.05	MUO	2.78 (1.69, 4.59)	< 0.0001	2.67 (1.61, 4.41)	< 0.01

*Note:* Model 1: Unadjusted. Model 2: Adjusted for age, gender, drinking, and smoking habits. Definition 1: Obesity defined by BMI. Definition 2: Obesity defined by WC instead of BMI. IDF criteria: Metabolic disorders defined by 2005 International Diabetes Federation criteria. CDS criteria: Metabolic disorders defined by 2013 Chinese Diabetes Society criteria.

Abbreviations: MHNO, metabolically healthy non‐overweight/obesity; MHO, metabolically healthy overweight/obesity; MUNO, metabolically unhealthy non‐overweight/obesity; MUO, metabolically unhealthy overweight/obesity; OR, odds ratio.

*p* value < 0.05 indicates significance.

### 3.4 Further In‐Depth Subgroup Analysis

After categorizing different metabolic abnormalities, we initially stratified the participants based on BMI, as shown in Supporting Information S1: Table S4. The findings revealed that individuals with hypertension, but without other metabolic anomalies, demonstrated a statistically significant positive correlation with CVD in both normal weight and overweight/obesity groups, regardless of whether the IDF‐2005 or CDS‐2013 criteria were applied. In cases with three combined metabolic abnormalities, a pronounced positive correlation with CVD was observed, regardless of the BMI status and the criteria used (all *p* < 0.01 or *p* < 0.0001). Moreover, when combining metabolic abnormalities, the association between CVD and overweight/obese individuals was stronger compared to that observed in those with normal weight. Subsequently, we used WC instead of BMI to define overweight/obesity, as shown in Supporting Information S1: Table S5. Among individuals with a single metabolic abnormality, those with hypertension positively correlated with CVD across all weight categories, regardless of the criteria used for metabolic disorders. The coexistence of hyperglycemia and dyslipidemia did not exhibit a statistically significant correlation with CVD in both criteria, regardless of WC status (all *p* > 0.05). In cases involving the combination of three metabolic abnormalities, a significant positive correlation with CVD was consistently observed, regardless of whether the subjects were overweight/obese or the criteria used (all *p* < 0.01 or *p* < 0.0001).

## 4 Discussion

In this study, we characterized the association between different metabolic overweight/obesity phenotypes and the prevalence of CVD. We found that both body weight and metabolic status affect the prevalence of CVD. In elderly population, WC was positively associated with higher CVD prevalence, rather than BMI. Hypertension presented as the strongest risk factor for CVD, highlighting the importance of antihypertensive therapy in CVD prevention. Previously, there was a lack of research on obesity phenotype in the Chinese population; our study addresses this gap.

Overweight and obesity is the major risk factor affecting the prevalence of CVD. Research demonstrated that obesity accelerates the process of atherosclerosis through insulin resistance, lipid oxidation, and vascular inflammation [27]. In our study, we used BMI and WC to define overweight/obesity simultaneously. WC is one of the simplest indicators, reflecting the accumulation of visceral abdominal fat. Nevertheless, the WC index lacks consideration for height measurements, potentially limiting its predictive capability for cardiovascular risk factors in both tall and short populations [28, 29]. In clinical settings, overweight and obesity are typically defined by BMI. However, research indicates that WC may be a better predictor of CVD compared to BMI [30, 31] because BMI fails to account for variations in adipose tissue distribution, which are closely linked to CVD.

Currently, multiple criteria have been developed to define metabolic syndrome, such as the US National Cholesterol Education Program (NCEP) criteria, the International Diabetes Federation (IDF) criteria, and the Chinese Diabetes Society (CDS) criteria. Hence, the prevalence of metabolic syndrome fluctuates within the same population due to variations in the defining criteria [32, 33]. In our study, IDF and CDS criteria were used for the analysis to mitigate the potential impact of utilizing different criteria on the outcomes. Difference was observed in the MHO group defined by the two criteria, probably due to the different cutoff point of BMI in defining overweight/obesity. Asian population had different association between BMI and body fat compared to the European population, and the proportion of Asian people with high risk of CVD were relatively lower than WHO cutoff point of overweight [34]. As a result, BMI ≥ 24 kg/m^2^ may be a better cutoff value in the definition of overweight/obesity in the Chinese population, in order to predict CVD risk. Moreover, the difference in our results between the two criteria also indicated research on MetS should focus on the variation brought by different definitions.

In this study, we found that MUNO and MUO groups were significantly associated with higher CVD prevalence compared to the MHNO group. In contrast, MHO participants had a lower risk of CVD compared to MUO and MUNO participants, indicating that MHO is an intermediate‐risk state [33, 35, 36]. However, MHO is not a static condition. Bobbioni‐Harsch et al. reported that, after 3 years of follow‐up, more than half of the subjects who are MHO at baseline developed more than one cardiometabolic risk factor [37]. Similarly, in the Whitehall II cohort study, about half of the initially MHO subjects converted to a metabolically unhealthy status after 20 years of follow‐up [38]. Similar results were also observed in the Chinese population [39]. Even though individuals with obesity who remain metabolically healthy still have a higher risk of developing CVD compared to MHNO subjects, according to the Nurses' Health Study [40]. MUNO individuals were often characterized by lower lipid storage capacity, lower percentage leg fat mass, more visceral and liver fat content, impaired adipose tissue function, and lower insulin sensitivity compared to MHO [41]. Some studies showed that MUNO individuals were associated with a higher risk of CVD compared to MHO individuals [42, 43], which is consistent with our results. As expected, compared to MUNO individuals, MUO individuals exhibited a stronger association with CVD, highlighting the need for more intensive treatment of metabolic abnormalities in the overweight/obese population.

CVDs are more prevalent in middle‐aged and older adults, especially in those aged 50 and older. In our study, the mean age of the participants was 55.89 ± 8.03 years, which suggested that our results provide a relatively strong representation of the association between obesity phenotype and CVD. Then, we divided the included population into subgroups by age. In the 60–80 age group, when BMI was used to identify obesity, MUO was significantly associated with CVD. However, when using WC, both obesity and metabolic unhealthy phenotypes showed significant association with CVD, which indicated that WC may serve as a better predictor of CVD in elderly compared to BMI. Several changes of body composition are observed in the elderly: loss of body height, decrease in muscle mass, and increase in fat mass, especially in abdominal visceral and intramuscular fat mass, without obvious change in BMI [44, 45]. In a systematic review and meta‐analysis, Ellen and colleagues reported that elevated WC was associated with higher CVD–related mortality in the elderly, regardless of BMI values [46]. In a prospective cohort study involving 418 adults aged ≥ 60, higher WC was associated with increased CVD mortality [47]. Thus, for the elderly, we should pay more attention on WC, rather than BMI.

Interestingly, our study found that patients with hypertension were significantly associated with a higher prevalence of CVD, regardless of whether they were obese or non‐obese. Hypertension is the one of the most important and independent risk factors of CVD, with a high prevalence of exposure. In US population, hypertension accounts for more atherosclerotic cardiovascular disease (ASCVD) death than other ASCVD risk factors [48]. Ramezankhani, Azizi, and Hadaegh reported that among components of metabolic syndrome, only elevated blood pressure and high fasting plasma glucose were significantly linked to an increased risk of CVD, noting that managing hypertension could reduce the risk of CVD over a 10‐year follow‐up period [49]. In a study by Khalili and colleagues, a latent class analysis based on MetS components involving 2598 middle‐aged participants revealed that the hypertension class was the only significant predictor of CVD [50]. This is consistent with prior research [51, 52], which underscores the critical role of antihypertensive therapy in preventing CVD. Moreover, when combined with hypertension, overweight or obese individuals showed stronger association with CVD, indicating that greater emphasis should be placed on blood pressure management within the overweight or obese population.

Some limitations should be noted in our study. First, given the cross‐sectional design, it is not possible to establish causal relationships between metabolic obesity phenotypes and CVD. Future studies should further clarify the risks between different metabolic phenotypes and CVD. Second, since the history of CVD in our study was based on self‐reports, this may introduce information bias. Third, our sample only included subjects aged 40–80 years, which may limit the applicability of our findings to younger populations. Fourth, the gender ratio of our study population was imbalanced. Therefore, future studies should aim to recruit a more balanced number of male and female participants to improve the interpretation of the results.

## 5 Conclusion

In conclusion, our study found that both obesity and metabolic unhealthiness were associated with a higher prevalence of CVD, indicating that body weight and metabolic status are both risk factors for CVD. Older individuals should pay more attention on maintaining a healthy WC rather than solely focusing on BMI. Moreover, hypertension showed the strongest correlation with CVD. Therefore, clinical interventions should focus on both body weight management and the treatment of metabolic disease simultaneously, especially anti‐hypertensive therapy. Additionally, individuals with hypertension, especially when they are overweight or obese, should pay more attention to early screening for CVD. In the future, more studies on overweight/obesity metabolic phenotypes should be performed so as to provide more precise therapeutic strategy in clinical practice.

## Author Contributions

Meng Ren and Shujin Fan conceived and designed the experiments. Yue Qiu and Shujin Fan wrote this manuscript. Jing Liu and Yue Qiu obtained and organized the clinical data. Shujin Fan and Xiaodan He analyzed the data. Tianxin Zhu and Li Yan checked and revised the manuscript. All authors read and approved the final manuscript. We thank everyone who helped in completing this study.

## Ethics Statement

This study was approved by the Ethics Committee of Sun Yat‐sen Memorial Hospital, Sun Yat‐sen University. The ethics approval number is Sun Yat‐sen Memorial Hospital of Zhongshan University [2019] Ethical Approval Research No. 38.

## Consent

All authors approved this manuscript to be published.

## Conflicts of Interest

The authors declare no conflicts of interest.

## Supporting information

Supporting information.

## Data Availability

The data sets generated and/or analyzed during the current study are available in the REACTION study.
