# Supplementary material for: Association Between Overweight/Obesity Metabolic Phenotypes Defined by Two Criteria of Metabolic Abnormality and Cardiovascular Diseases: A Cross‐Sectional Analysis in a Chinese Population
Source: Clin Cardiol. 2024 Oct 14;47(10):e70020. doi: 10.1002/clc.70020 (PMC11473791; doi:10.1002/clc.70020)
Supplement: Supplementary file 1 — Supporting information. [file CLC-47-e70020-s001.docx]

**Supplement Table 1 Criteria of metabolic abnormalities with IDF-2005 and CDS-2013**

| **Risk factors** | **IDF-2005 criterion** | **CDS-2013 criterion** |
| --- | --- | --- |
| Dyslipidemia | TG ≥ 1.7 mmol/L or/and  HDL-c:  men < 40 mg/dL (1.03 mmol/L);  women < 50 mg/dL (1.29 mmol/L)  or treatment for this lipid abnormality | TG ≥ 1.7 mmol/L or/and HDL-c:  < 40 mg/dL (1.03 mmol/L) or treatment for this lipid abnormality |
| Hypertension | Systolic ≥ 130 mmHg or/and  Diastolic ≥ 85 mmHg  or treatment of previously diagnosed hypertension | Systolic: ≥ 130 mmHg or/and  Diastolic: ≥ 85 mmHg  or treatment of previously diagnosed hypertension. |
| Hyperglycemia | fasting plasma glucose (FBG) ≥ 5.6 mmol/L, or/and previously diagnosed type 2 diabetes | fasting plasma glucose (FBG) ≥ 6.1 mmol/L or/and OGTT-2h ≥ 7.8 mmol/L,  or previously diagnosed type 2 diabetes |

**Supplement Table 2** **Baseline characteristics of different metabolic phenotypes defined by IDF criteria and CDS criteria in definition 1**

| **IDF-2005 criteria** | MHNO | MHO | MUNO | MUO | P value | **CDS-2013 criteria** | MHNO | MHO | MUNO | MUO | P value |
| --- | --- | --- | --- | --- | --- | --- | --- | --- | --- | --- | --- |
| N | 3773 | 903 | 2208 | 1841 |  |  | 3252 | 1745 | 1624 | 2104 |  |
| Age, years | 54.47 ± 7.40 | 54.01 ± 7.51 | 58.27 ± 8.20 | 56.71 ± 8.36 | <0.001 |  | 54.38 ± 7.32 | 54.14 ± 7.12 | 58.48 ± 8.32 | 57.52 ± 8.57 | <0.001 |
| Waist circumference,cm | 77.58 ± 7.47 | 87.66 ± 7.29 | 80.89 ± 7.54 | 90.54 ± 7.58 | <0.001 |  | 76.49 ± 7.10 | 86.35 ± 7.48 | 79.79 ± 7.53 | 89.42 ± 7.57 | <0.001 |
| Body mass index, kg/m2 | 22.00 ± 1.72 | 27.16 ± 3.26 | 22.71 ± 1.56 | 27.54 ± 2.68 | <0.001 |  | 21.58 ± 1.47 | 26.31 ± 2.89 | 22.12 ± 1.38 | 26.79 ± 2.67 | <0.001 |
| Systolic blood pressure, mmHg | 118.24 ± 12.70 | 120.66 ± 10.74 | 134.86 ± 16.00 | 135.75 ± 16.27 | <0.001 |  | 117.95 ± 12.61 | 122.22 ± 12.96 | 134.91 ± 16.15 | 136.33 ± 16.07 | <0.001 |
| Diastolic blood pressure, mmHg | 71.63 ± 8.30 | 72.90 ± 6.68 | 78.71 ± 9.83 | 80.90 ± 10.14 | <0.001 |  | 71.38 ± 8.28 | 74.36 ± 8.65 | 78.47 ± 9.72 | 80.56 ± 9.97 | <0.001 |
| Fasting glucose, mmol/L | 5.21 ± 0.83 | 5.26 ± 0.73 | 6.25 ± 1.62 | 6.22 ± 1.61 | <0.001 |  | 5.28 ± 0.86 | 5.37 ± 0.86 | 6.13 ± 1.70 | 6.26 ± 1.65 | <0.001 |
| Total cholesterol, mmol/L | 1.22 ± 0.68 | 1.23 ± 0.51 | 1.90 ± 1.43 | 2.20 ± 1.63 | <0.001 |  | 1.16 ± 0.63 | 1.32 ± 0.71 | 1.92 ± 1.40 | 2.25 ± 1.66 | <0.001 |
| HDL cholesterol, mmol/L | 1.45 ± 0.35 | 1.40 ± 0.31 | 1.17 ± 0.33 | 1.15 ± 0.30 | <0.001 |  | 1.46 ± 0.34 | 1.37 ± 0.31 | 1.19 ± 0.35 | 1.14 ± 0.31 | <0.001 |
| OGTT-2h, mmol/L | 7.02 ± 2.33 | 7.30 ± 2.04 | 9.24 ± 3.55 | 9.36 ± 3.33 | <0.001 |  | 6.78 ± 2.18 | 6.95 ± 2.07 | 9.64 ± 3.36 | 9.92 ± 3.33 | <0.001 |
| Gender, % |  |  |  |  | <0.001 |  |  |  |  |  | <0.001 |
| Female | 2814 (74.58%) | 700 (77.52%) | 1469 (66.53%) | 1275 (69.26%) |  |  | 2467 (75.86%) | 1346 (77.13%) | 1057 (65.09%) | 1388 (65.97%) |  |
| Male | 959 (25.42%) | 203 (22.48%) | 739 (33.47%) | 566 (30.74%) |  |  | 785 (24.14%) | 399 (22.87%) | 567 (34.91%) | 716 (34.03%) |  |
| Cardiovascular diseases, % |  |  |  |  | <0.001 |  |  |  |  |  | <0.001 |
| No | 3713 (98.41%) | 881 (97.56%) | 2130 (96.47%) | 1758 (95.49%) |  |  | 3203 (98.49%) | 1703 (97.59%) | 1575 (96.98%) | 2001 (95.10%) |  |
| Yes | 60 (1.59%) | 22 (2.44%) | 78 (3.53%) | 83 (4.51%) |  |  | 49 (1.51%) | 42 (2.41%) | 49 (3.02%) | 103 (4.90%) |  |
| Drinking, % |  |  |  |  | 0.69 |  |  |  |  |  | 0.158 |
| No | 2846 (76.48%) | 681 (76.00%) | 1692 (77.58%) | 1385 (76.27%) |  |  | 2470 (77.09%) | 1291 (74.67%) | 1244 (77.70%) | 1599 (76.88%) |  |
| Yes | 875 (23.52%) | 215 (24.00%) | 489 (22.42%) | 431 (23.73%) |  |  | 734 (22.91%) | 438 (25.33%) | 357 (22.30%) | 481 (23.12%) |  |
| Smoking, % |  |  |  |  | 0.002 |  |  |  |  |  | <0.001 |
| No | 3287 (88.36%) | 813 (91.04%) | 1875 (86.25%) | 1594 (87.29%) |  |  | 2837 (88.71%) | 1553 (89.92%) | 1365 (85.15%) | 1814 (87.00%) |  |
| Yes | 433 (11.64%) | 80 (8.96%) | 299 (13.75%) | 232 (12.71%) |  |  | 361 (11.29%) | 174 (10.08%) | 238 (14.85%) | 271 (13.00%) |  |

Definition 1: obesity defined by body mass index. IDF-criteria: metabolic disorders defined by 2005 International Diabetes Federation criteria. CDS-criteria: metabolic disorders defined by 2013 Chinese Diabetes Society criteria. MHNO: metabolic healthy non-overweight/obesity; MHO: metabolic healthy overweight/obesity; MUNO: metabolic unhealthy non-overweight/obesity; and MUO: metabolic unhealthy overweight/obesity. OGTT-2h: Oral Glucose Tolerance Test 2-Hour; HDL: high-density lipoprotein. All data are reported as mean ± SD and absolute frequency where proper. P value < 0.05 indicates significant.

**Supplement Table 3** **Baseline characteristics of different metabolic phenotypes defined by IDF criteria and CDS criteria in definition 2**

| **IDF-2005 criteria** | MHNO | MHO | MUNO | MUO | P value | **CDS-2013 criteria** | MHNO | MHO | MUNO | MUO | P value |
| --- | --- | --- | --- | --- | --- | --- | --- | --- | --- | --- | --- |
| N | 3238 | 1951 | 1589 | 2297 |  |  | 4118 | 1180 | 2188 | 1589 |  |
| Age, years | 54.45 ± 7.49 | 54.61 ± 7.45 | 57.67 ± 8.07 | 57.77 ± 8.57 | <0.001 |  | 54.24 ± 7.23 | 54.99 ± 7.59 | 57.82 ± 8.21 | 58.18 ± 8.90 | <0.001 |
| Waist circumference,cm | 74.42 ± 6.36 | 87.12 ± 6.94 | 77.94 ± 6.34 | 89.66 ± 7.49 | <0.001 |  | 75.82 ± 6.35 | 91.02 ± 6.56 | 79.33 ± 5.72 | 92.84 ± 6.73 | <0.001 |
| Body mass index, kg/m^2^ | 21.56 ± 2.64 | 25.02 ± 2.92 | 22.63 ± 2.59 | 26.10 ± 3.02 | <0.001 |  | 22.04 ± 2.78 | 25.91 ± 3.08 | 23.12 ± 2.53 | 26.79 ± 3.00 | <0.001 |
| Systolic blood pressure, mmHg | 117.93 ± 13.05 | 120.65 ± 12.80 | 134.06 ± 16.24 | 136.43 ± 15.73 | <0.001 |  | 118.36 ± 13.00 | 121.82 ± 12.53 | 134.96 ± 16.11 | 136.70 ± 16.08 | <0.001 |
| Diastolic blood pressure, mmHg | 71.40 ± 8.60 | 73.15 ± 8.20 | 78.86 ± 10.04 | 80.07 ± 9.87 | <0.001 |  | 71.72 ± 8.59 | 73.81 ± 8.40 | 78.92 ± 9.89 | 80.59 ± 9.88 | <0.001 |
| Fasting glucose, mmol/L | 5.21 ± 0.78 | 5.25 ± 0.90 | 6.20 ± 1.51 | 6.36 ± 1.70 | <0.001 |  | 5.29 ± 0.87 | 5.39 ± 0.86 | 6.11 ± 1.62 | 6.34 ± 1.76 | <0.001 |
| Total cholesterol, mmol/L | 1.16 ± 0.63 | 1.38 ± 0.81 | 1.84 ± 1.42 | 2.15 ± 1.60 | <0.001 |  | 1.16 ± 0.58 | 1.38 ± 0.86 | 1.99 ± 1.48 | 2.25 ± 1.64 | <0.001 |
| HDL cholesterol, mmol/L | 1.46 ± 0.35 | 1.40 ± 0.33 | 1.16 ± 0.34 | 1.16 ± 0.31 | <0.001 |  | 1.45 ± 0.34 | 1.36 ± 0.32 | 1.18 ± 0.35 | 1.14 ± 0.31 | <0.001 |
| OGTT-2h, mmol/L | 6.97 ± 2.26 | 7.34 ± 2.37 | 9.02 ± 3.41 | 9.62 ± 3.53 | <0.001 |  | 6.79 ± 2.17 | 7.03 ± 2.12 | 9.64 ± 3.29 | 10.03 ± 3.42 | <0.001 |
| Gender, % |  |  |  |  | <0.001 |  |  |  |  |  | <0.001 |
| Female | 2172 (67.08%) | 1707 (87.49%) | 846 (53.24%) | 1790 (77.93%) |  |  | 3090 (75.04%) | 940 (79.66%) | 1407 (64.31%) | 1078 (67.84%) |  |
| Male | 1066 (32.92%) | 244 (12.51%) | 743 (46.76%) | 507 (22.07%) |  |  | 1028 (24.96%) | 240 (20.34%) | 781 (35.69%) | 511 (32.16%) |  |
| Cardiovascular diseases, % |  |  |  |  | <0.001 |  |  |  |  |  | <0.001 |
| No | 3187 (98.42%) | 1906 (97.69%) | 1531 (96.35%) | 2196 (95.60%) |  |  | 4052 (98.40%) | 1145 (97.03%) | 2109 (96.39%) | 1514 (95.28%) |  |
| Yes | 51 (1.58%) | 45 (2.31%) | 58 (3.65%) | 101 (4.40%) |  |  | 66 (1.60%) | 35 (2.97%) | 79 (3.61%) | 75 (4.72%) |  |
| Drinking, % |  |  |  |  | <0.001 |  |  |  |  |  | 0.528 |
| No | 2388 (74.74%) | 1531 (79.33%) | 1150 (73.48%) | 1815 (79.92%) |  |  | 3098 (76.25%) | 903 (77.31%) | 1657 (76.75%) | 1226 (78.04%) |  |
| Yes | 807 (25.26%) | 399 (20.67%) | 415 (26.52%) | 456 (20.08%) |  |  | 965 (23.75%) | 265 (22.69%) | 502 (23.25%) | 345 (21.96%) |  |
| Smoking, % |  |  |  |  | <0.001 |  |  |  |  |  | <0.001 |
| No | 2729 (85.63%) | 1800 (93.12%) | 1273 (81.19%) | 2054 (90.48%) |  |  | 3591 (88.58%) | 1046 (89.63%) | 1845 (85.34%) | 1374 (87.24%) |  |
| Yes | 458 (14.37%) | 133 (6.88%) | 295 (18.81%) | 216 (9.52%) |  |  | 463 (11.42%) | 121 (10.37%) | 317 (14.66%) | 201 (12.76%) |  |

Definition 2: obesity defined by waist circumference instead of body mass index. IDF-criteria: metabolic disorders defined by 2005 International Diabetes Federation criteria. CDS-criteria: metabolic disorders defined by 2013 Chinese Diabetes Society criteria. MHNO: metabolically healthy non-overweight/obesity; MHO: metabolically healthy overweight/obesity; MUNO: metabolically unhealthy non-overweight/obesity; and MUO: metabolically unhealthy overweight/obesity. OGTT-2h indicates Oral Glucose Tolerance Test 2-Hour; HDL, high-density lipoprotein. All data are reported as mean ± SD and absolute frequence where proper. P value < 0.05 indicates significant.

**Supplement Table 4** **Association between CVD and different combinations of metabolic disorder defined by IDF criteria and CDS criteria, stratified by BMI.**

|  | OR (95% CI) | P value | OR (95% CI) | P value | OR (95% CI) | P value |  | | OR (95% CI) | P value | OR (95% CI) | | P value | | | OR (95% CI) | | P value |
| --- | --- | --- | --- | --- | --- | --- | --- | --- | --- | --- | --- | --- | --- | --- | --- | --- | --- | --- |
|  | **IDF-2005 criteria** | | | | | | | **CDS-2013 criteria** | | | |  | | |  | |  |  |
|  | Total |  | Normal Weight |  | Overweight/  obesity |  |  | | Total |  | Normal Weight | | |  | | Overweight/  obesity | |  |
| Metabolic healthy | 1 |  | 1 |  | 1 |  |  | | 1 |  | 1 | | |  | | 1 | |  |
| Single Hypertension | 3.19 (1.81, 5.62) | <0.0001 | 2.51 (1.29, 4.86) | <0.01 | 8.16 (1.82, 36.50) | <0.01 |  | | 3.69 (2.10, 6.51) | <0.0001 | 3.77 (1.73, 8.20) | | | <0.01 | | 3.49 (1.50, 8.10) | | <0.01 |
| Single hyperglycemia | 2.04 (1.07, 3.88) | <0.05 | 1.76 (0.85, 3.66) | 0.13 | 4.55 (0.87, 23.68) | 0.07 |  | | 3.12 (1.76, 5.52) | <0.0001 | 3.73 (1.79, 7.80) | | | <0.01 | | 2.42 (0.98, 5.98) | | 0.06 |
| Single dyslipidemia | 1.17 (0.63, 2.17) | 0.62 | 0.92 (0.44, 1.90) | 0.82 | 3.08 (0.65, 14.59) | 0.16 |  | | 1.03 (0.47, 2.28) | 0.94 | 1.28 (0.45, 3.66) | | | 0.64 | | 0.80 (0.24, 2.67) | | 0.71 |
| Hypertension with hyperglycemia | 3.14 (1.71, 5.78) | <0.01 | 3.77 (1.94, 7.32) | <0.0001 | 3.54 (0.68, 18.40) | 0.13 |  | | 5.82 (3.43, 9.87) | <0.0001 | 6.33 (3.09, 12.96) | | | <0.0001 | | 5.23 (2.37, 11.56) | | <0.0001 |
| Hypertension with dyslipidemia | 2.59 (1.45, 4.60) | <0.01 | 2.54 (1.29, 4.97) | <0.01 | 4.89 (1.09, 22.02) | <0.05 |  | | 3.20 (1.71, 6.02) | <0.01 | 4.70 (2.01, 10.98) | | | <0.01 | | 2.29 (0.90, 5.87) | | 0.08 |
| Hyperglycemia with dyslipidemia | 1.93 (1.05, 3.55) | <0.05 | 1.86 (0.91, 3.79) | 0.09 | 3.70 (0.79, 17.25) | 0.10 |  | | 2.30 (1.24, 4.24) | <0.01 | 2.08 (0.81, 5.32) | | | 0.13 | | 2.29 (0.96, 5.44) | | 0.06 |
| Hypertension with hyperglycemia and dyslipidemia | 5.18 (3.17, 8.48) | <0.0001 | 3.82 (2.15, 6.78) | <0.0001 | 12.75 (3.08, 52.72) | <0.01 |  | | 4.53 (2.69, 7.62) | <0.0001 | 3.43 (1.50, 7.84) | | | <0.01 | | 4.57 (2.15, 9.73) | | <0.0001 |

Definition 1: obesity defined by body mass index. IDF-criteria: metabolic disorders defined by 2005 International Diabetes Federation criteria. In IDF criteria, participants with a normal weight have a BMI ranging from 18.5 to 25 kg/m^2^, while overweight/obesity participants have a BMI ≥ 25 kg/m^2^. CDS-criteria: metabolic disorders defined by 2013 Chinese Diabetes Society criteria. In CDS criteria, participants with a normal weight have a BMI ranging from 18.5 to 24 kg/m^2^, while overweight/obesity participants have a BMI ≥ 24 kg/m^2^. OR: odds ratio. P value < 0.05 indicates significant.

**Supplement Table 5** **Association between CVD and different combinations of metabolic disorder defined by IDF criteria and CDS criteria, stratified by WC.**

|  | OR (95% CI) | P value | OR (95% CI) | P value | OR (95% CI) | P value |  | OR (95% CI) | P value | OR (95% CI) | P value | OR (95% CI) | P value |
| --- | --- | --- | --- | --- | --- | --- | --- | --- | --- | --- | --- | --- | --- |
|  | **IDF-2005 criteria** | | | |  |  |  | **CDS-2013 criteria** | | | |  |  |
|  | Total |  | Normal  Weight |  | Overweight/  obesity |  |  | Total |  | Normal  Weight |  | Overweight/obesity |  |
| Metabolic healthy | 1 |  | 1 |  | 1 |  |  | 1 |  | 1 |  | 1 |  |
| Single Hypertension | 2.75 (1.61, 4.68) | <0.01 | 2.25 (1.12, 4.53) | <0.05 | 3.76 (1.53, 9.23) | <0.01 |  | 3.51 (2.07, 5.96) | <0.0001 | 2.95 (1.52, 5.70) | <0.01 | 4.26 (1.64, 11.03) | <0.01 |
| Single hyperglycemia | 1.91 (1.05, 3.47) | <0.05 | 1.77 (0.84, 3.75) | 0.14 | 2.29 (0.82, 6.39) | 0.11 |  | 2.89 (1.70, 4.92) | <0.0001 | 3.29 (1.78, 6.06) | <0.01 | 2.01 (0.69, 5.86) | 0.20 |
| Single dyslipidemia | 1.04 (0.58, 1.86) | 0.90 | 0.65 (0.27, 1.56) | 0.34 | 1.65 (0.66, 4.11) | 0.28 |  | 1.00 (0.48, 2.11) | 0.99 | 0.85 (0.31, 2.30) | 0.75 | 1.15 (0.35, 3.81) | 0.82 |
| Hypertension with hyperglycemia | 2.86 (1.61, 5.07) | <0.01 | 2.95 (1.39, 6.27) | <0.01 | 3.17 (1.24, 8.14) | <0.05 |  | 5.28 (3.22, 8.66) | <0.0001 | 5.35 (2.94, 9.74) | <0.0001 | 4.82 (1.94, 11.99) | <0.01 |
| Hypertension with dyslipidemia | 2.45 (1.43, 4.19) | <0.01 | 2.62 (1.28, 5.35) | <0.01 | 2.71 (1.11, 6.58) | <0.05 |  | 2.90 (1.59, 5.31) | <0.01 | 3.56 (1.70, 7.46) | <0.01 | 2.09 (0.71, 6.08) | 0.18 |
| Hyperglycemia with dyslipidemia | 1.70 (0.95, 3.02) | 0.07 | 1.59 (0.71, 3.54) | 0.26 | 2.05 (0.81, 5.17) | 0.13 |  | 2.08 (1.16, 3.74) | <0.05 | 1.86 (0.85, 4.04) | 0.12 | 2.14 (0.80, 5.76) | 0.13 |
| Hypertension with hyperglycemia and dyslipidemia | 4.65 (2.94, 7.33) | <0.0001 | 4.25 (2.32, 7.79) | <0.0001 | 5.69 (2.58, 12.55) | <0.0001 |  | 4.25 (2.61, 6.91) | <0.0001 | 5.20 (2.87, 9.43) | <0.0001 | 3.30 (1.37, 7.97) | <0.01 |

Definition 2: obesity defined by waist circumference instead of body mass index. IDF-criteria: metabolic disorders defined by 2005 International Diabetes Federation criteria. In IDF criteria, participants with overweight/obesity were defined as WC ≥ 90 cm in men while ≥ 80 cm in women. CDS-criteria: metabolic disorders defined by 2013 Chinese Diabetes Society criteria. In CDS criteria, participants with overweight/obesity were defined as WC ≥ 90 cm in men while ≥ 85 cm in women. OR: odds ratio. P value < 0.05 indicates significant.
